# Inulin‐type fructans and short‐chain fructooligosaccharides—their role within the food industry as fat and sugar replacers and texture modifiers—what needs to be considered!

**DOI:** 10.1002/fsn3.3040

**Published:** 2022-09-15

**Authors:** Peter Philip James Jackson, Anisha Wijeyesekera, Robert Adrian Rastall

**Affiliations:** ^1^ Department of Food and Nutritional Sciences University of Reading Reading UK

**Keywords:** carbohydrates, chemistry, food processing, inulin, physical, polysaccharides, prebiotics, sensory

## Abstract

Inulin and oligofructose are classes of prebiotics belonging to a group of nondigestible carbohydrates referred to as inulin‐type fructans. While short‐chain fructooligosaccharides are enzymatically synthesized from the hydrolysis and transglycosylation of sucrose. Inulin‐type fructans and short‐chain fructooligosaccharides act as carbon sources for selective pathways supporting digestive health including altering the composition of the gut microbiota along with improving transit time. Due to their physicochemical properties, inulin‐type fructans and short‐chain fructooligosaccharides have been widely used in the food industry as partial replacements for both fat and sugar. Yet, levels of replacement need to be carefully considered as it may result in changes to physical and sensory properties that could be detected by consumers. Furthermore, it has been reported depending on the processing parameters used during production that inulin‐type fructans and short‐chain fructooligosaccharides may or may not undergo structural alterations. Therefore, this paper reviews the role of inulin‐type fructans and short‐chain fructooligosaccharides within the food industry as fat and sugar replacers and texture modifiers, their impact on final sensory properties, and to what degree processing parameters are likely to impact their functional properties.

## Graphical abstract

Inulin‐type fructans and short‐chain fructooligosaccharides are becoming and increasingly common ingredient used in the food industry as fat and sugar replacers and texture modifiers. Yet careful consideration must be given to the type and quantity of fructans used in replacement of fat and sugar in order product integrity while maintaining product quality.

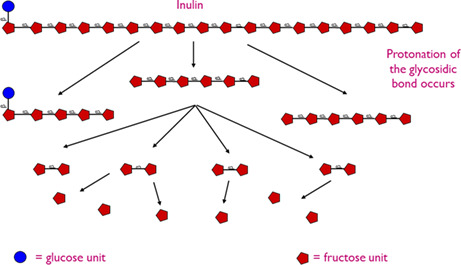

## 1 INTRODUCTION

With the incidence of obesity and obesity‐related diseases on the increase, the food industry is facing increasing pressure from both governmental and public health bodies to modify food products to reduce both fat and sugar intakes to help combat the burden of disease (Krystyjan et al., 2015; Rodriguez‐Garcia et al., 2013). Both fat and sugar play vital roles in determining the physical, chemical, and, ultimately, sensory properties of several food products enjoyed by consumers, so it has become necessary for manufacturers to find suitable alternatives to both fat and sugar that do not result in drastic changes to a given food products’ final rheological and sensory quality.

Inulin and oligofructose (OF) are nondigestible carbohydrates belonging to a group of carbohydrates termed inulin‐type fructans (ITF; Karimi et al., 2015). ITF are composed of monomers of fructose joined by β‐(2–1) glycosidic (fructosyl‐fructose) linkages with varying degrees of polymerization (DP; Mensink et al., 2015). ITF are predominantly extracted from plants with the DP depending on the source, time of year, and length of post‐harvest storage. For example, wheat, bananas, and onions possess short‐chain ITF (max DP < 10). Jerusalem artichokes possess medium‐chain ITF (max DP < 40) and globe artichokes and chicory root possess long‐chain ITF (max DP < 100; Roberfroid et al., 2010). In this regard, with the exception of chicory root, which possesses up to 70% inulin on a dry weight basis, most of these fruits and vegetables only possess trace amounts of ITF, and as a result, the production of ITF primarily focuses on chicory root (Mensink et al., 2015).

The production of ITF from chicory in principle consists of three major steps: (1) extraction via hot water; (2) purification to remove impurities, and finally, (3) spray drying. If required, inulin may undergo enzymatic hydrolysis to alter the DP resulting in the production of OF (Apolinario et al., 2014). The length of the fructose chain determines the physiochemical properties of ITF and therefore its uses in food, with differences in physiochemical properties between OF and inulin becoming increasingly apparent at DP > 10 (Roberfroid et al., 2010; Shoaib et al., 2016). Alternately, short‐chain oligofructose (scFOS) can be enzymatically synthesized by catalyzing the hydrolysis and transglycosylation of sucrose leading to the formation of 1‐kestose (Glu‐Fru2), 1‐nystose (Glu‐Fru3), and 1F‐β‐fructofuranosylnystose (Glu‐Fru4; Rastall, 2010).

OF and scFOS due to its shorter chain length and greater solubility combined with possessing a sweetness value of 30%–35% that of sucrose means OF and scFOS can be used as a partial replacement for sugar (Villegas et al., 2010). While in contrast, long‐chain ITF (LC‐ITF) due to their greater DP and resulting water‐binding properties can form fat‐mimicking gels at concentrations >10%–20% providing reduced fat foods with similar textural and sensory characteristics of full‐fat versions (Elleuch et al., 2011; Karimi et al., 2015).

Furthermore, given the resistance of ITF and scFOS to digestion due to the absence of brush boarder β‐fructosidases, they can act as excellent bulking agents due to their low calorific content (1–1.5 kcal/g) having the potential to reduce energy intake by 65%–75% compared to digestible carbohydrates (Soukoulis & Fisk, 2016). This also means that ITF and scFOS reach the colon intact, functioning as a prebiotic for beneficial microorganisms within the gut (bifidobacteria are the group most frequently targeted), providing health benefits to the host summarized as in these series of reviews (Ahmed & Rashid, 2019; Sanders et al., 2019; Wilson & Whelan, 2017).

Due to these abilities, ITF and scFOS have been used in the production of a range of different food products including cakes, muffins, bread, ready‐to‐eat breakfast cereals, cheese, ice cream, yogurt, fruit juices, and even Lyon‐style sausages among others with a great deal of success (Bi et al., 2016; Di Criscio et al., 2010; Klewicki, 2007; Peressini et al., 2015; Rodriguez‐Garcia et al., 2013; Salazar et al., 2009). However, it is common for food products to be subjected to multiple different processes during production including baking (Poinot et al., 2010), pH adjustment (Klewicki, 2007), extrusion (Tsokolar‐Tsikopoulos et al., 2015), and even high‐pressure pasteurization, all of which have the potential to alter the physiochemical properties of ITF.

Given the development of functional and low‐fat and sugar food products aiming to improve and support gut and overall health, this area shows no sign of slowing down anytime soon. In addition, the number of people willing to spend a premium on products that they believe to be good for their health is seemingly on the increase (Karelakis et al., 2019; Vicentini et al., 2016). Therefore, the purpose of this review is to explore the role of ITF and scFOS within the food industry as fat and sugar replacers and viscosity modifiers, along with identifying how various food processing parameters may potentially alter ITF physiochemical integrity.

## 2 THE PHYSIOCHEMICAL PROPERTIES AND EFFECTS OF FOOD PROCESSING ON THE STRUCTURAL INTEGRITY OF INULIN‐TYPE FRUCTANS AND SHORT‐CHAIN FRUCTOOLIGOSACCHARIDES

ITF and scFOS, in molecular terms, can be divided into two subgroups based on their DP with distinct physiochemical properties. The distinction between these two groups has a critical cut‐off point of around DP 10 (van Loo, 2006).

### 2.1 Solubility

The solubility of ITF is closely related to DP with solubility decreasing with increasing chain length. In aqueous solution at room temperature, short‐chain ITF (DP < 10) and scFOS are highly soluble at around 80% (w/w; van Loo, 2006). While in contrast, the solubility of medium and LC‐ITFs varies greatly dependent on the DP of the molecule in question. On this basis, Orafti® GR (granulated inulin powder ‐ DP > 10) has a solubility of around 10% while Orafti® HP (inulin powder for fat replacement at low temperature ‐ DP > 23) has low solubility and Orafti® HSI (a highly soluble inulin powder) possesses high solubility (Moser & Wouters, 2014). The solubility of ITF is also somewhat dependent on temperature with the solubility of even LC‐ITF increasing as temperatures rise. This was demonstrated using LC‐ITF (DP > 23) at temperatures ranging from 50 to 90°C where it was possible to achieve a solubility of between 20 and 34% (Cui et al., 2013; Kim et al., 2001). Clearly, while temperature does appear to increase the solubility of LC‐ITF, it does not reach that of OF and scFOS and DP is the most critical factor regarding solubility.

### 2.2 pH

**FIGURE 1:**
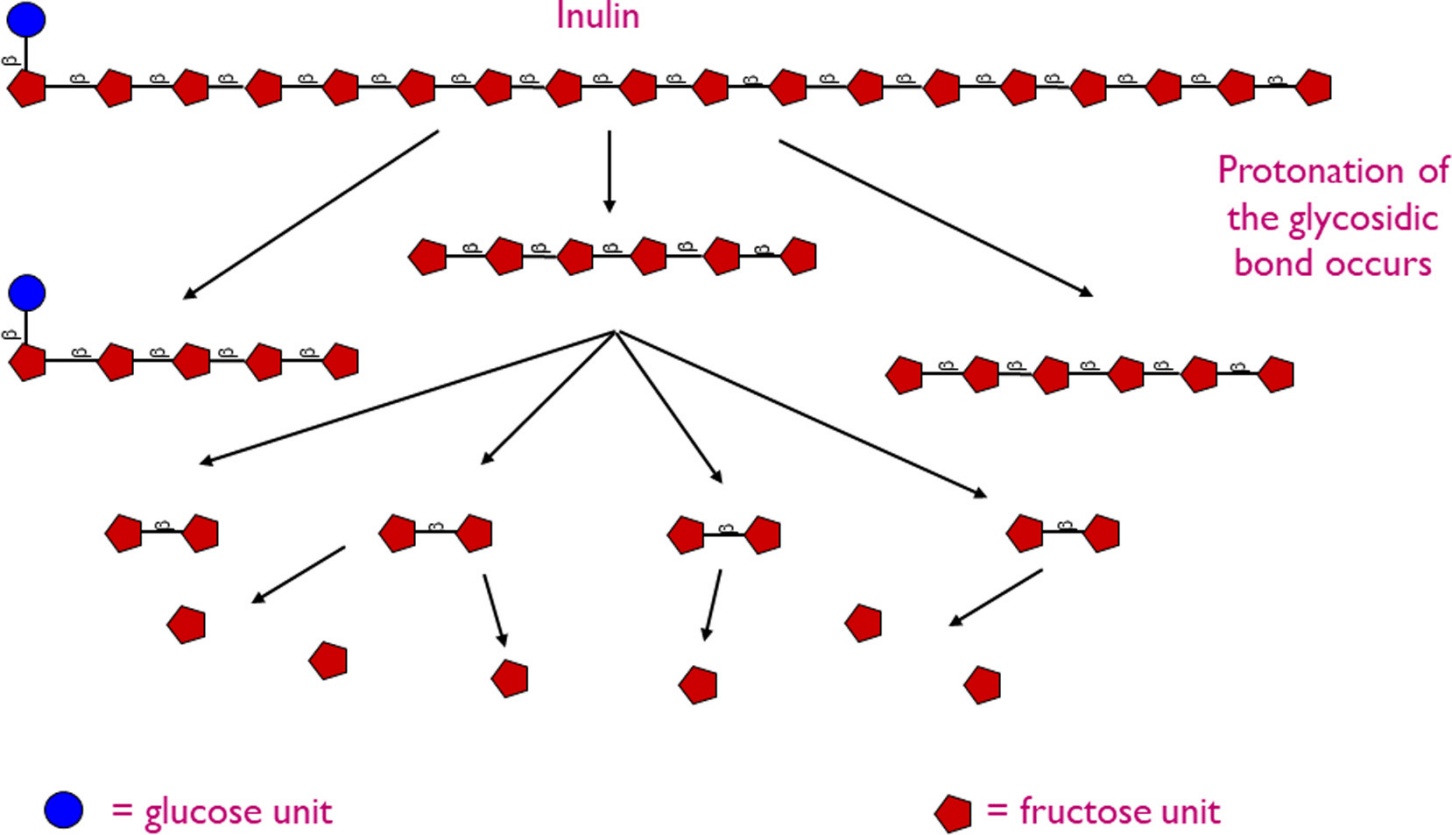
Hydrolysis of inulin results in the formation of shorter chains OF, fructose monomers, and a smaller amount of glucose monomers

The β‐(2,1) linkages of ITF and scFOS can be degraded by acid hydrolysis resulting in the production of OF and fructose leading to a reduction in nutritional properties (Glibowski & Wasko, 2008; Mensink et al., 2015; Figure 1). The hydrolysis of ITF and scFOS is pH dependent with a little‐to‐no breakdown of ITF at pH >4, while at pH <4, ITF are hydrolyzed. This is likely due to the protonation of the glycosidic bond (Blecker et al., 2002; Duar et al., 2015; Klewicki, 2007).

The hydrolysis of ITF and scFOS follows first‐order kinetics with inulin and OF/scFOS reporting similar trends concerning pH, temperature, and molecular weight (Barclay et al., 2012). Yet, rates of hydrolysis do vary between LC‐ITF and OF/scFOS due to differences in kinetics. The rates of hydrolysis of LC‐ITF are markedly slower than OF and scFOS, to begin with, due to the scarcity of end‐chain fructosyl groups (Mensink et al., 2015) along with higher activation energy required to stimulate hydrolytic cleavage implying that hydrolysis of mid‐chain glycosidic linkages in long‐chain ITF is greater (Barclay et al., 2012). As rates of hydrolysis of mid‐chain glycosidic linkages increase, rate of reaction subsequently increases due to the greater number of end‐chain fructosyl groups able to participate in the reaction (Blecker et al., 2002).

Yet, it is possible to control the rate at which hydrolysis occurs by altering the conditions or the DP of the ITFs used during production or storage. For example, by keeping the pH >4 hydrolysis and/or employing LC‐ITF, hydrolysis can be significantly reduced even as temperature and time increase up to 100°C for 55 min regardless of the thermal process used (Glibowski & Bukowska, 2011; Matusek et al., 2009).

### 2.3 Thermal processing

ITF and to a lesser extent scFOS are widely used as functional ingredients in several food products, including bread, biscuits, and cakes, where, depending on the process used, they may be subject to thermal degradation. However, while the thermal stability of ITF has been studied, due to several confounding factors results between studies are often hard to compare. For example, in the direct heating of inulin with temperatures ranging from 135 to 195°C for 5–60 min, the most critical times and temperatures for ITF degradation appeared to occur at 165°C for 30 min and 195°C for 15 min, respectively (Bohm et al., 2005). While, in contrast, Huebner et al. (2008) noted that ITF were functionally stable when heated in solution at 85°C for up to 6 h at neutral pH (pH 7). However, neither of these studies reflects the matrices of a more complex food product.

A more realistic example of the heating of fructans was undertaken by (Whelan et al., 2011) with the authors analyzing the fructan content of several supermarket loaves of bread as well as white bread vs. white toast. The authors noted after correction for loss of moisture that white toast recorded a lower fructan content compared to its white bread counterpart (0.28 vs. 0.33 g/slice). However, while these results suggest toasting results in a small loss of fructan content, the amount lost (0.05 g) is possibly not of functional significance.

**FIGURE 2:**
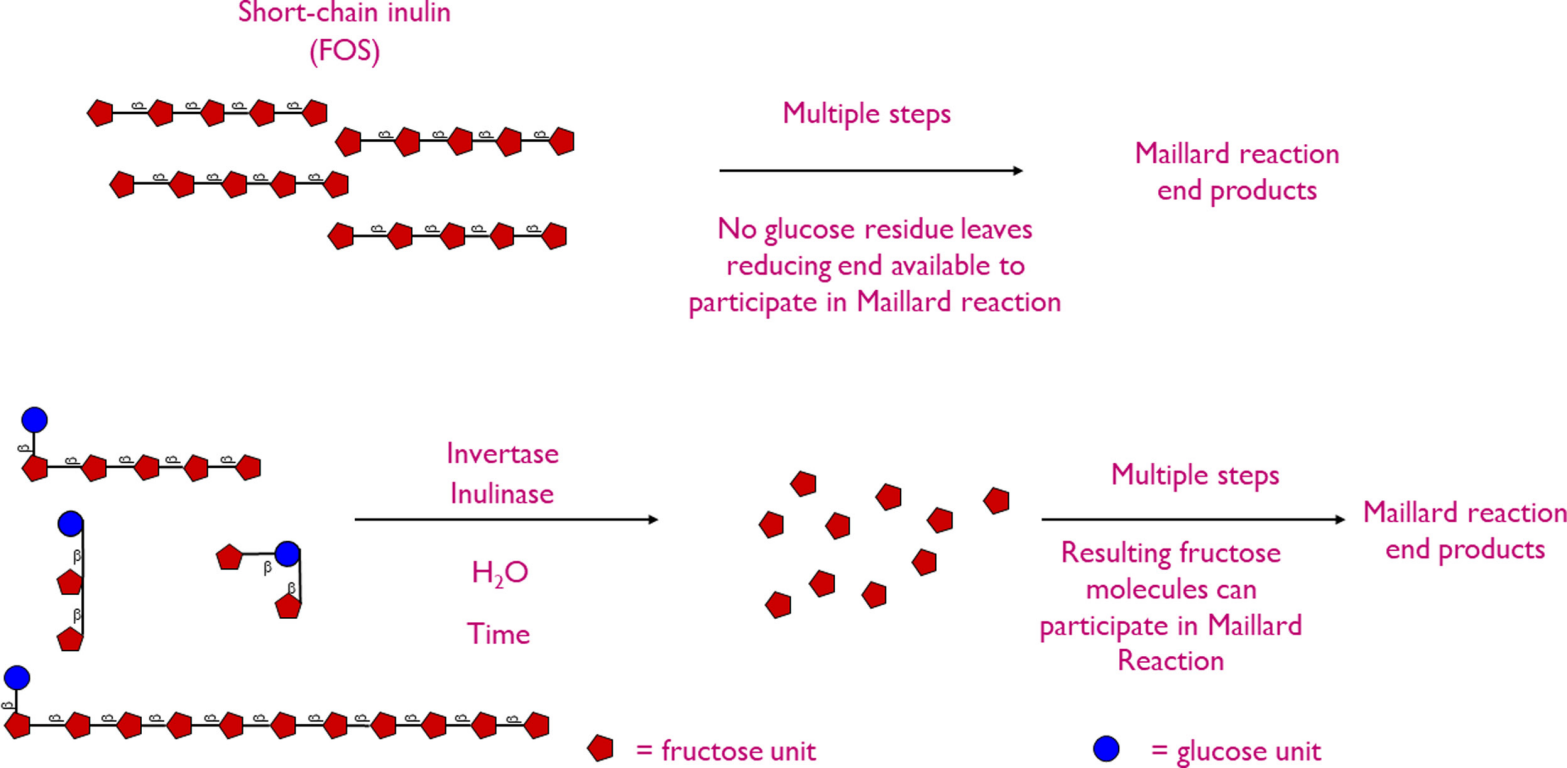
Pathways by which inulin‐type fructans and scFOS can participate in the Maillard reaction. Path A: Direct participation in the Maillard reaction due to the availability of the reducing end. Pathway B via the enzymatic effects of yeasts, invertase, and inulinase.

The formation of brown pigments in toast and baked products occurs as a result of the Maillard reaction, a nonenzymatic reaction between the amino group of amino acids and the carbonyl group of reducing sugars (Lund & Ray, 2017). ITF is a mixture of reducing and nonreducing oligosaccharides, therefore is susceptible to participation in the Maillard reaction (Mensink et al., 2015). Figure 2 summarizes the way fructans may participate in the Millard reaction.

The influence of the baking process on the color of ITF‐fortified baked products has been investigated to a substantial degree in bread (Poinot et al., 2010), sponge cakes (Rodriguez‐Garcia et al., 2012), muffins (Zahn et al., 2010), and shortbread biscuits (Leiva‐Valenzuela et al., 2018). The consensus of these studies is that the addition of ITF alters the color (level of browning) of the final product. Furthermore, Poinot et al. (2010) noted that bread containing 5% ITF darkened 3 min quicker than loaves of bread possessing 0 and 3% ITF. This suggests that reducing ITF do undergo Maillard browning, accelerating the rate of baking. However, as most baked products are only cooked for a short amount of time with the internal temperature of the food matrix not reaching more than 100°C, the rate of Maillard reaction associated with ITF degradation is likely to be minimal.

### 2.4 Yeast and enzyme degradation

**FIGURE 3:**
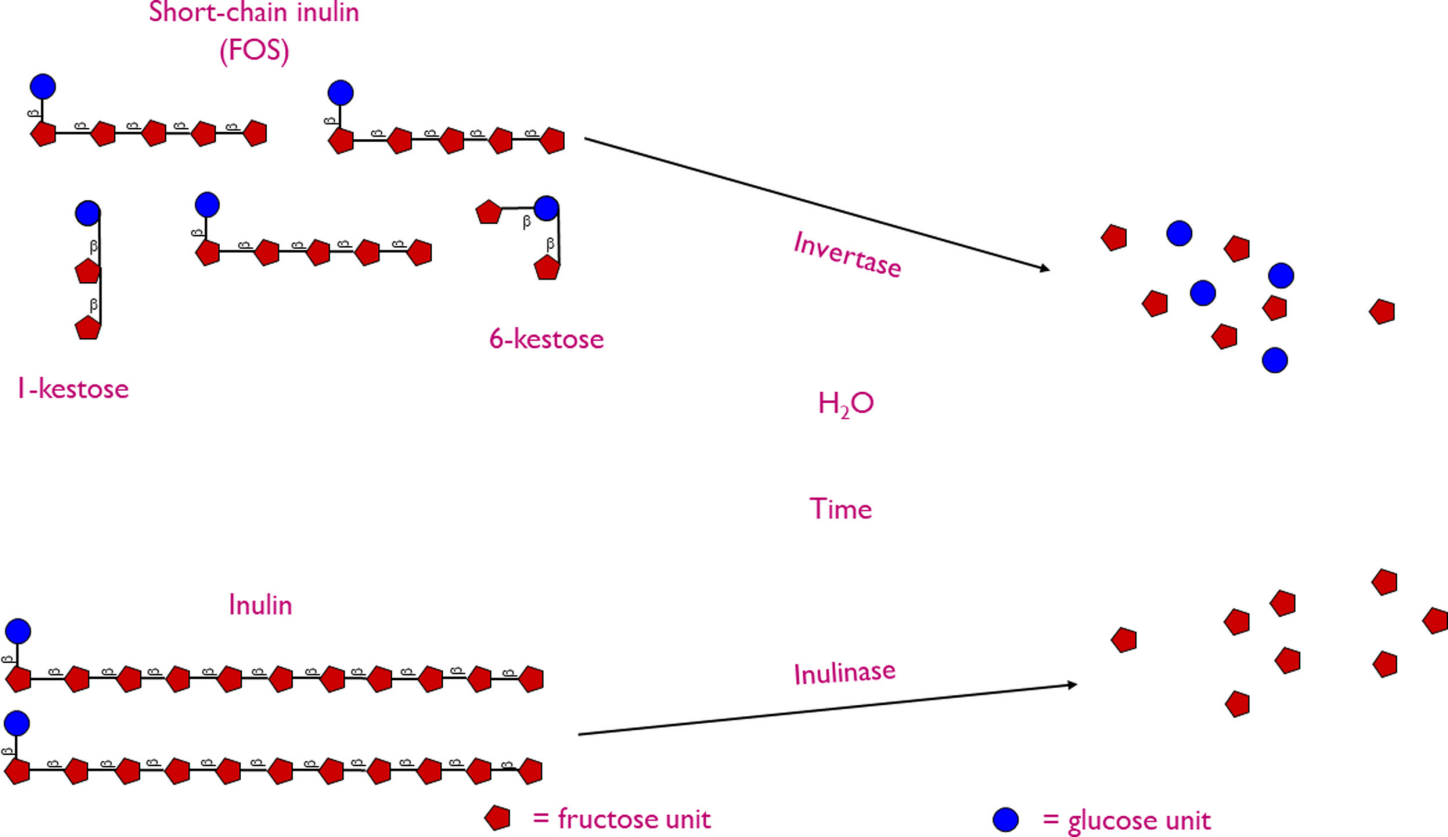
The degradation of inulin‐type fructans and scFOS via invertase and inulinase, respectively.

The bread‐making process is highly complex involving mixing, bulk fermentation, knocking back, proofing, shaping, and baking (Struyf, Van der Maelen, et al., 2017). During fermentation, yeasts produce several enzymes, including invertase and inulinase (Struyf, Van der Maelen, et al., 2017). Invertase primarily not only targets the α‐β‐(1–2) linkage of sucrose hydrolyzing sucrose into its respective monosaccharides, fructose, and glucose but can also target the β‐(2–1) glycosidic (fructosyl‐fructose) linkages of ITF, scFOS, and wheat‐type fructans (Struyf, Laurent, et al., 2017). On the other hand, inulinase can hydrolyze sucrose but shows higher specificity toward β‐(2–1) glycosidic (fructosyl‐fructose) linkages of fructans compared to invertase primarily due to the lack of chain‐end fructosyl groups (Menezes et al., 2018). Given enough time, up to 80%–90% of non‐ITF/ITF and scFOS can be degraded (Morreale et al., 2019; Verspreet et al., 2013; Figure 3). This leaves the reducing fructose and glucose available for both fermentation and the Maillard reaction, probably contributing toward more rapid browning recorded by Poinot et al. (2010).

The rate of hydrolysis of ITF/scFOS appears to be somewhat dependent on the type of yeast used during production, with commercial and wild bread/beer strains tending to show a higher degree of fructan degradation compared to low‐/noninvertase‐producing yeasts (Fraberger et al., 2018; Gelinas et al., 2016; Morreale et al., 2019; Verspreet et al., 2013). This suggests that the rate of ITF/non‐ITF degradation during bread making may be reduced by using low‐invertase‐producing yeasts. However, low‐invertase‐producing yeasts are not readily available to the bread‐making industry. A more realistic alternative may be to alter the type of yeast and fructans used, with (Rakha et al., 2010) indicating that *S. cerevisiae* invertase has a preference for low‐DP fructans (<5). This suggests that the degradation of fructans could be minimized by employing LC‐ITF (DP > 10) during the production of bread.

### 2.5 High temperature and pressure extrusion

Extrusion is a process where several foods, including wholegrain and cereal slurries, are subjected to a combination of different temperatures, pressures, times, and shear rates (Offiah et al., 2019). This process results in alterations to the physical structure of the whole grain cereals, transforming them into puffed ready‐to‐eat food products including breakfast cereals and snacks (Peressini & Sensidoni, 2009; Sacchetti et al., 2005; Tsokolar‐Tsikopoulos et al., 2015).

For a ready‐to‐eat extruded breakfast cereal or snack to be well perceived, the final product should possess low density and a high degree of porosity and expandability (Bisharat et al., 2013). These properties result in a feeling of lightness and an increased perception of crispness which are considered ideal properties by the consumer (Tsokolar‐Tsikopoulos et al., 2015).

Extrusion cooking has become an increasingly common technology used in the food processing industry as it allows manufacturers to fortify food products with additives including vitamins, minerals, and dietary fibers which may have been lost in other parts of the production process (Korkerd et al., 2016). Therefore, it should come as no surprise that the addition of ITF and scFOS to extruded food products has been extensively studied (Brennan et al., 2008; Capriles et al., 2009; Tsokolar‐Tsikopoulos et al., 2015).

On this basis, Brennan et al. (2008) investigated the effect of the addition of ITF at 5%, 10%, and 15% on the characteristics of ready‐to‐eat breakfast cereals. The authors noted that increasing the level of ITF increased the level of crispness. However, despite the addition of ITF improving the crispness of ready‐to‐eat breakfast cereals as ITF, fortification increased from 5%–10%–15% expandability decreased. Yet, even at 10% and 15% ITF fortification expandability was not statistically different compared to the non‐ITF control. This finding is consistent with that reported by Tsokolar‐Tsikopoulos et al. (2015) who noted that as ITF fortification increased hardness also increased, while the expansion ratio decreased. As the level of porosity is linked to cell wall rupture (Tsokolar‐Tsikopoulos et al., 2015), the addition of ITF to starchy products results in increases in cell wall rupture reducing the expandability of air bubbles, subsequently increasing density and hardness (Anton et al., 2009). Any shortcoming in expandability can be overcome to a certain degree by increasing temperature and/or screw speed. However, significant adjustments in processing parameters may result in alterations to the functional properties of ITF. This was demonstrated by Duar et al. (2015) who reported that under several different extrusion conditions, including 140°C at a screw speed of 170 rpm more than 50% of scFOS was degraded. Yet, LC‐ITF appeared to be unaffected by low‐temperature (120°C) extrusion. However, when screw speeds were adjusted to 120 and 170 rpm, only 25% and 34% of LC‐ITF were recovered. Furthermore, only low levels of LC‐ITF were recovered (35%) when temperature and screw speed were operated at their most extreme conditions: temperature (170°C) and pressure (170 rpm), respectively.

A more realistic way to overcome any losses of ITF/scFOS during the extrusion process may be to add inulin into extruded cereals and snacks via the flavoring process. Under this premise, Capriles et al., (2009) demonstrated that the addition of 13.3% inulin and OF added via the flavoring process to extruded snacks produced a final product with no significant differences in sensory attributes. It seems clear that ITF can provide a realistic avenue for functional fortification in both extruded snacks and ready‐to‐eat breakfast cereals.

## 3 THE ROLE OF INULIN‐TYPE FRUCTANS AND SCFOS IN THE FOOD INDUSTRY

### 3.1 Inulin‐type fructans and short‐chain fructooligosaccharides as fat replacers

Fat, particularly saturated fat from animal sources, is a significant contributor to the development of obesity and obesity‐related diseases including cardiovascular disease (CVD; Dickson‐Spillmann & Siegrist, 2011; Vasilopoulou et al., 2020). As a result, there have been ever‐increasing demands placed on manufacturers to reformulate products to produce low‐fat alternatives (La Berge, 2008). Replicating the beneficial properties that fat plays in the final quality of food products is, however, highly complex due to its multifunctional properties. Fat not only contributes to taste, flavor, and caloric intake but it can also inhibit gluten formation providing ideal snap and spread in baked products (Rodriguez‐Garcia et al., 2012; Rodriguez‐Garcia et al., 2013; Rodriguez‐Garcia et al., 2014) along with enhancing both the mouthfeel and melting rates of dairy products including cheese, chocolate, and ice cream (Tekin et al., 2017; Wadhwani et al., 2011). Inulin, in particular LC‐ITF, due to their physiochemical properties, can form fat‐like gels at >10%–20%. Yet, replacing fat inulin does pose several challenges which are discussed below. Table 1 summarizes these findings.

**TABLE 1:** Summarized findings of ITF and scFOS used as fat replacers in food products

Reference	Food product	Type of inulin	Level of fat/fructan replacement/addition	Outcomes/findings
Akbari et al., 2016	Ice cream	Fruitfit®TEX, molecular weight 3300, average DP > 20	80% fat to 8% of the final composition	Lower hardness values were detcted in ITF fortified low‐fat ice cream compared to non‐ITF low‐fat ice cream, but higher hardness values compared to full‐fat control (*p <* .05) 4% containing low‐fat ITF cream produced similar sensory scores compared to full‐fat control
Tiwari et al., 2015	Ice cream	No details	2%, 4%, and 6%	Melting rates increased significantly (*p* < .05) with ITF substitution At 2% and 4% ITF‐fat replacement, no noticeable differences in sensory characteristics (appearance, flavor, body, and texture were detected). At 6% ITF‐fat replacement, sensory characteristics were less acceptable across all test parameters
Hashemi et al., 2015	Ice cream	Orafti®HP (High Performance) inulin, average DP > 23	5%	Higher levels of overrun, viscosity, and hardness values compared to the full‐fat control No differences in taste, texture, color, and mouthfeel compared to full‐fat control (all *p <* .05)
Guven et al., 2005`	Yogurt	Raftiline®HP, average DP > 23	1%, 2%, and 3%	At 1% ITF addition, separation of whey increased. Whey separation was not statically different at 2% and 3% ITF addition compared to full‐fat control (*p* > .05) At 1, 2, and 3% ITF addition, no differences in body or texture were detected (*p* < .05) At 2% and 3% ITF addition, significant different in term of consistency compared to whole‐milk yogurt (*p* < .05)
Pimentel et al., 2012	Yoghurt	Raftiline®HP, average DP > 23	2%	Levels of whey separation increased (*p* < .05) No differences in levels of firmness, cohesiveness, adhesiveness, and gumminess were detected compared to skimmed and full‐fat control (*p* > .05)
Brennan & Tudorica, 2008	Yoghurt	Frutafit®HD, average DP 8–13 (9)	2%, 4%, and 6%	At 2% ITF additon, no significant differences were detected in firmness, degree of whey separation, creaminess, and mouthfeel compared to both full‐fat and skimmed controls (*p* > .05) At 6% ITF addition, no differences were detected in firmness, creaminess, and mouthfeel scores compared to full‐fat and skimmed controls (*p* > .05) At 6% ITF addition, degree of whey separation was significantly greater compared to both full‐fat and skimmed controls (*p* > .05)
Kip et al., 2006	Yoghurt	Frutafit® HD, average DP ⩾9	1.5%, 3%, and 4%	As levels of levels of ITF concentrations increased levels of firmness and viscosity increased
Guggisberg et al., 2009	Yoghurt	Long‐chain Inulin (Pacovis, Stetten, Switzerland), average DP > 23	0%–4% (Fat 0.2%–3.5%)	The addition of ITF in low‐fat yogurt (0.1%, 1%, and 2% fat) at 2% visually reduced syneresis Levels of firmness and viscosity were not significantly impacted by different levels of ITF concentrations The creaminess was not impacted by either level of fat or inulin (*p* = .062 and *p* = .065) respectively. Creaminess did trend toward an increase with increasing fat and inulin levels. Creaminess perception was highly influenced by increasing concentrations of inulin and fat
Gonzalez et al., 2011	Peach‐flavored yogurt drink	Raftilose®P95, average DP < 10	14 g/L (1.4%)	No differences were detected in overall acceptability between whole‐ and skimmed‐milk peach‐flavored yogurt drinks containing ITF (*p* > .05). Whole‐milk ITF containing peach‐flavoured yoghurt drinks were generally more acceptable than their skimmed milk cointerparts
Borges et al., 2019	Sheep's milk cheese	Orafti®ST‐Gel, average DP < 10	50 g/L	Texture parameters (firmness, adhesiveness, cohesiveness, elasticity, and gumminess) were comparable to that of both whole milk and skimmed controls
Giri et al., 2017	Cheese spread	Frutafit®HD, average DP 8–13 (⩾9)	0%, 4%, 6%, and 8%	At up to 6% ITF addition, spreadability was comparable to the control. At 8% ITF addition spreadability decreased significantly (*p* < .05) Compared to control, color scores for all inulin cheeses were significantly lower (*p* < .05) At 8% ITF addition, spreadable cheese recorded significantly higher levels of hardness, coarseness, and spreabability (*p* < .05)
Hennelly et al., 2006	Cheese	Rafteline®HP, average DP > 23	5 g/100 g of inulin gel (54IN1) 13.75 g/100 g of inulin gel (54IN2), 13.75 g/100 g of inulin solution (54HIN; All moisture content 54 g/100 g) 13.13 g/100 g of inulin gel with a moisture content of 56 g/100 g was also manufactured (56MC)	ITF replacement in 56MC cheese resulted in significantly greater meltability compared to all other ITF‐replaced cheeses as well as the 54MC control (*p* < 0.05). Hardness values were significantly greater in all ITF‐replaced cheeses with the exception of 56MC compared to the 54MC control (all *p* < .05)
Solowiej et al., 2015	Casein processed cheese	Raftaline®HPX, average DP > 23	1%, 2%, and 3% (1, 2, and 3 g/100 g)	ITF at 1%, 2%, and 3% decreased hardness values compared to anhydrous milk fat control (all *p* < .05). Cheeses containing 15% or 20% anhydrous milk fat and 2%, and 3% ITF recorded higher melting values (*p* < .05)
Miocinovic et al., 2011	Low‐fat unfiltered milk cheese	Inulin (Cosucra, Belgium) Fibruline®—no specific details	1%, 1.5%, 2%, and 3%	Improvement in sensory characteristics with increasing I content, in particular mouthfeel
Li et al., 2019	Reduced‐fat mozzarella cheese‐like product	Inulin Shandong Futian Pharmaceutical Co., Ltd (Qingdao, China)—no specific details	2% (20 g/kg)	ITF‐enriched reduced‐fat cheese was considerably less rubbery, less firm, sweeter, and fattier than the noninulin‐containing reduced‐fat control mozzarella cheese
Rodriguez‐Garcia et al., 2013)	Shortbread biscuits	Frutafit® HD, average DP 8–13 (9)	10%, 20%, 30%, and 40%	At 20% and 30% fat replacement, panelists could differentiate between the 0 and 20% fat‐/ITF‐replaced biscuits
Krystyjan et al., 2015	Shortbread biscuits	Frutafit® IQ, average DP 8–12	20%, 30%, 40%, and 50%	At 20% and 30% fat replacement, no differences in sensory characteristics compared to the control were detected. At 40 and 50%, a reduction in sensory characteristics was observed
Rodriguez‐Garcia et al., 2012	Sponge cake	Frutafit® HD, average DP 8–13 (9)	0%, 35%, 50%, 70%, and 100%	At 50% and 70% fat replacement, colour, appearance, taste, texture, and overall acceptability were not statically signifiacnt comapred to the 0% inulin control (*p* > .05). Decline in batter viscosity, cake height, increases in chewiness and hardness were detected at 100% fat replacement (*p* < .05)
Rodriguez‐Garcia et al., 2014	Sponge cake	Frutafit HD®, average DP 8–13 (9)	50%	Loss of batter viscosity and less and more broader bubble distribution. At 50% fat replacement, no noticeable differences in sensory characteristics (appearance, color, texture, and taste) were detected (*p* > .05)
Zahn et al., 2010	Muffins	Color, average DP 10. Fibruline®S20, average DP < 10 Orafti®GR, average DP > 10	50%, 75%, and 100%	Batter flowability increased with an increasing amount of fat, which was substituted Moisture content increased in final muffins—10% for Fibruline® S20 and Orafti®GR, and c. 20% for Fibruline® Instant Muffins replaced with 50% in presented higher surface gloss, greater browning, and sweetness At 75% and 100% inulin replacement, crumb firmness significantly increased as well as glossiness and toughness (*p* < .05)
Mendoza et al., 2001	Dry‐fermented sausage	Raftiline®ST, average DP > 10	7.5% and 12.5%	Low‐ and medium‐fat sausages supplemented with ITF at 7.5% scored significantly greater scores for spiciness, tenderness, and softness compared to the full‐fat control (*p* < .05) Low‐ and medium‐fat ITF sausages supplemented with ITF at 7.5% were significantly harder compared to the full‐fat control (*p* < .05)
Mendez‐Zamora et al., 2015	Frankfurter Sausage	Orafti®GR, average DP > 10	Low fat with 15% inulin; low fat with 7.5% inulin and 7.5% pectin; low fat with 30% inulin; and low fat with 15% inulin and 15% pectin	Addition of ITF at 15% and 30% recorded lower lightness values (*p* < .05) At 30% fat/ITF replacement, the sausages scored lower in terms of hardness, gumminess, chewiness, flavor, and overall acceptance compared (*p* < .05)
Keenan et al., 2014	Sausage	Orafti® GR and Orafti® HP, average DP > 10 and >23	18.70% (DP ≥ 23) or 9.35% (DP ≥ 23) and 9.35% (DP ≥ 10)	The addition of ITF at 18.70% (DP ≥ 23) or 9.35% (DP ≥ 23) and 9.35% (DP ≥ 10) reduced the cooking loss (degree of shrinkage) The addition of ITF resulted in slight increases in hardness and chewiness values
Afshari et al., 2015	Burgers	Frutafit®TEX, average DP > 23	4% and 8%	Burgers possessing 4% and 8% ITF were significantly harder compared to the non‐ITF control (*p* < .05) Burgers containing 8% ITF possessed significantly higher levels of hardness compared to the 4% ITF burgers (*p* < .05) No significant differences in terms of overall acceptability were detected between the control and both 4% and 8% ITF‐containing burgers
Salazar et al., 2009	Dry‐fermented sausage	Actiligh®950P	2%, 4%, and 6% at 6%, 15%, and 30% backfat	The addition of scFOS to fermented sausages reduced the hardness of dry‐fermented sausages The addition of scFOS reduced gumminess, chewiness, and hardness, in particular at 6% supplementation at all levels of back fat
Caceres et al., 2004	Hot cooked sausage	Actilight®950P scFOS (GFn, *n* ⩽ 4) composed of a mix of 1‐kestose, nystose, and 1‐F‐fructofuranosyl nystose	2%, 4%, 6%, 8%, 10%, and 12% without fat reduction 2%, 4%, 6%, 8%, 10%, and 12% with 40% fat reduction	Non‐fat reduced and reduced fat sausages supplemented with scFOS recorded higher color values at 12% supplementation (*p* < .05) The addition of scFOS in non‐fat reduced sausages reduced hardness, chewiness, and gumminess (*p* < .05) Fat‐reduced, scFOS‐supplemented sausages were able to match the fat‐reduced, non‐scFOS‐supplemented sausage in terms of textural properties Both non‐fat and fat‐reduced scFOS‐supplemented sausages scored higher in terms of juiciness, but lower in terms of tenderness (*p* < .05)

### 3.2 Inulin‐type fructans and short‐chain fructooligosaccharides as fat replacers in dairy products

#### 3.2.1 Ice cream

Ice cream is a highly complex multiphase food product consisting of fat, sugar, protein, air bubbles, and ice crystals, all dispersed in a semifrozen solution (Akbari et al., 2019). On average, ice cream contains between 10 and 16% fat which affects both the physical and sensory properties of the product including smoothness, melting rate, aroma, flavor, aeration, and creaminess (Mahdian & Karazhian, 2013; Tekin et al., 2017). Of all ITF, LC‐ITF (DP > 23) represent the most realistic alternative to fat in ice cream due to their excellent gel‐forming properties, mimicking the mouthfeel quality found in high‐quality dairy‐based ice cream desserts (Gonzalez‐Tomas et al., 2009).

The effects of replacing 80% of fat (8% of the final composition) with ITF (DP > 20) at 2%, 3%, and 4% on the physicochemical properties and sensory attributes of low‐fat ice cream were investigated by (Akbari et al., 2016). The authors reported all low‐fat ITF‐containing ice creams recorded lower hardness values compared to the ITF‐free low‐fat ice cream but were all still significantly harder compared to the full‐fat control (*p* < .05). Yet, the low‐fat ice cream containing 4% ITF recorded similar sensory scores compared to the full‐fat control. Additionally, both (Tiwari et al., 2015; Hashemi et al., 2015) noted in ice cream in which fat had been replaced with 2%, 4%, and 5% ITF that no noticeable differences in sensory characteristics with the exception of hardness values were recorded compared to the high‐fat control. However, when ITF/fat replacement was increased to 6%, Tiwari et al. (2015) recorded noticeable differences in appearance, flavor, body, and texture.

The ability of ITF to alter the hardness, melting rates, and sensory characteristics of low‐fat ice cream results from several interacting factors, including altering the stability and composition of the fat crystal network (Muse & Hartel, 2004). LC‐ITF water‐binding capacity and gel‐forming properties modify the rheology of ice cream due to their ability to form microcrystals, which bind water molecules trapping them within the dispersed phase (Aykan et al., 2008; Karaca et al., 2009; Tiwari et al., 2015). This likely increase in the amount of unfrozen water found in the dispersed phase not only leads to a reduction in levels of hardness but also the rate of ice crystal formation resulting in depression of the final freezing point (Tiwari et al., 2015).

Furthermore, as the concentration of fat begins to decrease, the level of overrun begins to decline, resulting in a reduction in the number of fat globule clusters present, leading to a decline in the abundance of air bubbles which can be trapped within the matrices. This subsequently increases the rate of heat transfer and the melting rate of ice cream which has been associated with an improvement in sensory properties compared to low‐fat non‐ITF‐containing ice cream (Akbari et al., 2016; Muse & Hartel, 2004). Based on the sensory properties of ice cream, it appears that replacing fat with up to 5% ITF produces no detectable differences in product quality.

#### 3.2.2 Yogurt

Yogurt is one of the most highly consumed dairy products worldwide, frequently being consumed multiple times a day, and is a known source of several vitamins and minerals including calcium, iodine, and vitamin B12 (Moore et al., 2018). Yogurt represents one of the most promising areas of fructan fortification (Shoaib et al., 2016) due to its potential to prevent textural losses from fat reduction while also increasing/decreasing fiber and saturated fat intake simultaneously (Kleniewska et al., 2016). In addition, yogurt presents the opportunity to produce a symbiotic product potentially providing additional health benefits to the consumer (Moghadam et al., 2019).

All types of ITF have been utilized in the production of yogurt including OF, medium‐chain, and LC‐ITF (Guggisberg et al., 2009; Guven et al., 2005; Paseephol et al., 2008). Guven et al. (2005) investigated the effects that the addition of HP‐ITF (average DP > 23) at 1%, 2%, and 3% had on the rheological and sensory properties of low‐fat yogurt. The authors noted that at 1%, the separation of whey appeared to increase, while at 2% and 3% ITF, the degree of whey separation was not statistically significantly different compared to the full‐fat control (*p* > .05). Furthermore, when fat was reduced and replaced with 1%, 2%, and 3% ITF, no statistical differences in body and texture scores were found, however, only the low‐fat yogurt with 1% inulin concentration could match the whole‐milk yogurt in terms of consistency. These results are similar to those documented by (Pimentel et al., 2012) who noted that the addition of HP‐ITF at 2% improved the textural properties of skimmed‐milk yogurt similar to that of the whole‐milk yogurt control. In contrast (Brennan & Tudorica,^2008^; Kip et al., 2006) observed that higher additions of LC‐ITFs and ITF at 6% and 3% improved the viscosity and textural aspect (firmness, creaminess, and smoothness) of low‐fat yogurt in comparison to the low‐fat control. This is consistent with data reported by Paseephol et al. (2008) who noted that low‐fat yogurt supplemented with 4% HP‐ITF produced rheological behavior comparable to that of the full‐fat control yogurt. However, Guggisberg et al. (2009) demonstrated that while the addition of ITF to low‐fat yogurts (2% and 3.5% fat) improved levels of creaminess when fat was reduced to 0.2% and ITF increased to 4%, it was not possible to produce a yogurt with the same level of consistency and creaminess as the whole‐milk sample. Finally, Gonzalez et al. (2011) documented that peach‐flavored drinking yogurt made with nonfat dried milk and supplemented with OF at 1.4% (14 g/kg) was not significantly different from the control, scoring similar in terms of aroma, color, and mouthfeel, although whole‐milk samples possessing OF were generally more liked than their skimmed‐milk counterparts.

The level of firmness and extent to which whey separation occurs in ITF‐fortified low‐fat yogurt results from the concentration and type of ITF used as well as the levels of milk proteins present. As the concentrations of LC‐ITF increase above 4%, the presence of a secondary inulin network may partly hinder the formation of the protein network (Guggisberg et al., 2009), likely increasing the level of syneresis detected. Yet at between 2% and 4%, the addition of LC‐ITFs appears to enhance the mouthfeel and consistency of low‐fat yogurt by increasing milk gel strength via the formation of electrostatic, hydrogen, and hydrophobic bonds with both whey and casein proteins without altering the milk gel structure. This produces a more viscous final product, offsetting any reduction in milk gel strength seen by decreasing fat concentration (Arango et al., 2013; Krivorotova et al., 2017). This suggests that, while higher levels of ITF‐supplementation in yogurt may indeed be possible, up to 4% ITF (w/w) replacement for fat appears to be a reasonable target to aim for (Meyer et al., 2011).

#### 3.2.3 Cheese

The production of cheese has occurred for centuries and fat plays several functions in the physical, textural, and sensory properties of cheese (Karimi et al., 2015). However, due to increasing demands by consumers for low‐fat products, the production of low‐fat hard and soft cheese has escalated in recent years (Johansen et al., 2011). The manufacture of low‐fat cheese is considered a challenge as lowering fat content results in adverse changes in rheological and sensory characteristics (Bi et al., 2016; Sanchez‐Macias et al., 2012). These adverse changes include a more dense cheese as well as a chewy and rubbery matrix, poor melting qualities, lack of flavor, and off‐putting color (Diamantino et al., 2014; Rogers et al., 2010). To date, inulin, specifically ITF, has been used in the production of a variety of low‐fat cheeses including mozzarella, parmesan, cream, cottage, and spreadable cheeses.

In the production of these cheeses, HP inulin and LC‐ITF are the preferred inulin of choice due to their greater DP (>23) and therefore better gel‐forming properties (Arcia et al., 2011), and they have been shown to improve the mouthfeel, flavor, and spreadability of low‐fat soft and spreadable cheese (Borges et al., 2019; Giri et al., 2017). Additionally, the ability of ITF (Orafti HP®) to replace fat in imitation cheese has also been explored, with (Hennelly et al., 2006) concluding that ITF can replace up to 63% of fat (3.44 g/100 g cheese) in imitation cheese, albeit presenting a slight decrease in the honeycomb structure.

Similarly, Solowiej et al. (2015) demonstrated that inulin (Raftaline®HPX) could replace milk fat at 1%–3% in processed cheese analogs while also improving functional properties including increasing meltability and decreasing hardness and adhesiveness. This finding mimicked that found by (Miocinovic et al., 2011), with the results indicating that the addition of inulin (no specific details) at 1.5% improved the texture, as well as the functional properties of low‐fat cheese produced from ultrafiltered milk. Furthermore, Li et al. (2019) also studied the addition of inulin (no specific details) to a model reduced‐fat mozzarella cheese‐like product at 20 g/kg (2%). The authors concluded that despite not being able to exactly match the textural properties of the full‐fat control, the inulin‐enriched reduced‐fat cheese was considered less rubbery, less firm, sweeter, and fattier than the noninulin‐containing reduced‐fat control mozzarella cheese. These data suggest that inulin can be used as a partial fat replacer in low‐fat soft, spreadable, and imitation cheese without drastic changes in sensory attributes being detected.

### 3.3 Inulin‐type fructans and short‐chain fructooligosaccharides as fat replacers in baked goods

Baked goods including cakes and biscuits represent a complex system of foams and emulsions. In baked products, fat acts as a leavening agent, inhibiting gluten formation, providing tenderness, and contributing toward spread (Rodriguez‐Garcia et al., 2012; Zahn et al., 2010) along with adding moistness, occlusion of air bubbles, and providing structural stability (Krystyjan et al., 2015; Matsakidou et al., 2010). As with ice cream and yogurt, the partial or complete replacement of fat in baked products represents a significant challenge as it can result in significant alterations to the quality of the final product including influencing the perceived levels of crispness, taste, snap, and flavor along with altering shelf life (Blonska et al., 2014).

In baked products, several attempts over the years have been undertaken to try and partially replace fat with suitable alternatives including the viscosity modifiers and gelling agents xanthan, guar, and gellan gum (Colla et al., 2018; Kohajdova & Karovicova, 2009), as well as dietary fibers including ITF and scFOS (Rodriguez‐Garcia et al., 2012; Zahn et al., 2010). Of all these potential fat replacers, ITF have attracted considerable attention as not only can they act as creaming agents, viscosity modifiers, emulsifying agents, and stabilizers but also provide a much needed source of fiber (Rodriguez‐Garcia et al., 2012; Rodriguez‐Garcia et al., 2013).

#### 3.3.1 Biscuits

The snap, crumbliness, and richness are the principal qualities of biscuits by which they are judged (Blonska et al., 2014; Krystyjan et al., 2015; Rodriguez‐Garcia et al., 2013). Fat not only acts as a flavor enhancer but also as a lubricant, reducing dough stickiness and inhibiting gluten formation allowing for optimal biscuit spread to occur during baking, resulting in the production of thinner more delicate biscuits (Laguna et al., 2012; Pareyt et al., 2009).

The ability of fructans, especially ITF, to act as a fat replacer in shortbread biscuits was investigated by (Blonska et al., 2014; Krystyjan et al., 2015; Rodriguez‐Garcia et al., 2013) with fat/ITF replacement ranging from 9.3% to 50%. At 20 and 30% fat replacement with ITF, Rodriguez‐Garcia et al. (2013) and Krystyjan et al. (2015) recorded that, while panelists could differentiate between the 0% and 20% fat‐/ITF‐replaced biscuit and the full‐fat–fat control, they could only describe slight differences between samples. Yet, Krystyjan et al. (2015) recorded that when fat/ITF replacement increased to 50%, differences in sensory qualities of shortbread biscuits became increasingly apparent, recording lower scores for taste, color, aroma, shape, and consistency and higher levels of dough stickiness and hardness.

At 50% fat/ITF replacement, the lower levels of fat present in the final dough allow greater accessibility of flour components, such as gluten, to water (Krystyjan et al., 2015; Mamat & Hill, 2014). This is likely to contribute toward the stickier dough and sensory alterations detected by (Krystyjan et al., 2015), as fat/ITF replacement increased to 50%. Nevertheless, this aside, it appears that replacing 20%–30% fat with ITF can deliver a quality short dough biscuit with desirable sensory characteristics.

#### 3.3.2 Cakes and muffins

In contrast to biscuits, sponge cake and muffins are judged on their levels of softness, lightness, moistness, and cohesiveness of the sponge (Rodriguez‐Garcia et al., 2012; Rodriguez‐Garcia et al., 2014; Zahn et al., 2010). Sponge cakes contain on average between 15% and 30% fat (w/w) and this is critical for trapping air bubbles, acting as a leavening agent, along with providing moisture and tenderizing the crumb (Matsakidou et al., 2010).

In sponge cakes and muffins, the replacement of fat with ITF has been investigated extensively (Rodriguez‐Garcia et al., 2012; Rodriguez‐Garcia et al., 2014; Zahn et al., 2010), investigated the effects of replacing 50%, 75%, and 100% of margarine (10%–20% of the final batter) in muffins with various types of ITF‐possessing DP ranging from <10 to >10. The authors noted that low‐fat muffins replaced with 50% of either ITF were comparable to the full‐fat control, only presenting slight differences in crumb firmness, final cake volume, and height. However, as the replacement of fat with ITF increased from 75% to 100%, greater differences in these sensory properties became increasingly apparent. Additionally, Rodriguez‐Garcia et al. (2012) noted that cakes made with 50% and 70% ITF (average DP 8–13) were not statistically different in terms of color, appearance, taste, texture, and overall acceptability (*p* > .05). Yet, significant differences in batter viscosity and decline in cake height were detected along with alterations in sensory attributes, including increased hardness and chewiness values as the replacement of fat with ITF increased to 100% (*p* < .05).

Regarding the differences detected in batter viscosity and cake height, ITF must be dispersed in water before creaming leading to the presence of additional levels of free water in the matrices, subsequently reducing batter viscosity (Psimouli & Oreopoulou, 2013). A lack of batter viscosity leads to a reduction in the amount of air that can be occluded during the creaming process (Rodriguez‐Garcia et al., 2012; Zahn et al., 2010) with a subsequent loss during the baking process. This results in a lack of batter expansion likely leading to the losses in cake height detected by both (Zahn et al., 2010) and (Rodriguez‐Garcia et al., 2012). Yet, it must be emphasized that reduction in sensory properties only becomes apparent when fat replacement with ITF exceeds 50%–70%. Fat replacement in cakes and muffins up to 50%–70% (w/w) with ITF can be achieved without significant losses in sensory properties.

### 3.4 Inulin‐type fructans and short‐chain fructooligosaccharides as fat replacers in meat and pate‐style salamis

Besides dairy and baked products, both ITF and scFOS have also been used as fat replacers in Frankfurter, Lyon‐style, and dry‐fermented sausages, as well as beef burgers (Afshari et al., 2015; Keenan et al., 2014; Mendoza et al., 2001). Mendoza et al. (2001) investigated the effects of replacing backfat with varying levels of ITF, on the sensory properties of dry‐fermented sausages. The results showed the addition of inulin at 7.5% improved the spiciness, tenderness, and softness of both medium and low‐fat ITF‐fortified sausages. However, both the medium‐ and low‐fat ITF‐fortified sausages could not quite match the juiciness of the conventional, high‐fat control.

In another study, Mendez‐Zamora et al. (2015) recorded that the replacement of fat with ITF at 30% improved both the yield and color of Frankfurter‐style sausages, however, at 30% fat/ITF replacement, the sausages scored lower in term of hardness, gumminess, chewiness, flavor, and overall acceptance compared to the full‐fat Frankfurter control. Yet, when fat was substituted with ITF at 15%, no differences in sensory characteristics were detected. Additionally, Keenan et al. (2014) concluded that the inclusion of ITF in sausages at 18.70% (DP ≥ 23) or 9.35% (DP ≥ 23) and 9.35% (DP ≥ 10) not only reduced the cooking loss (degree of shrinkage) in the sausages but also resulted in textural modifications including slight increases in the levels of hardness and chewiness detected. This finding is similar to those recorded by Afshari et al. (2015) who concluded that low‐fat burgers containing ITF at 8% had not only the highest levels of fat retention but also the highest levels of hardness and gumminess.

In addition to inulin‐type fructans, scFOS has also been used as fat replacers in dry‐fermented sausages (Salazar et al., 2009). In this study, scFOS was incorporated at 2%, 4%, and 6% into sausages containing 30%, 15%, and 6% backfat. The authors noted that the addition of scFOS to fermented sausages reduced hardness with dry‐fermented sausages at 15% fatback being the most accepted samples. However, the addition of scFOS did appear to result in a loss of color (lightness) due to increased turbidity likely due to scFOS gel‐forming properties.

Furthermore, another study (Caceres et al., 2004) investigated the effects that scFOS had on the sensory characteristics of cooked sausages either with or without fat reduction (40%) with ScFOS being supplemented at 2%, 4%, 6%, 8%, 10%, and 12%. Unsparingly cooked sausage with and without fat reduction and the addition of scFOS specifically at 12% produced higher color values (*p* < .05). While the addition of scFOS in nonfat‐reduced sausages reduced hardness, chewiness, and gumminess, the fat‐reduced scFOS‐supplemented sausages were able to match the non‐scFOS fat reduced control sausages in terms of textural properties. More interestingly, in terms of sensory characteristics, both nonfat‐ and fat‐reduced and scFOS‐supplemented sausages scored higher in terms of juiciness, but lower in terms of tenderness (*p* < .05). No other differences were detected with the scFOS‐supplemented sausages being considered acceptable by the panelists.

The basis of the textural changes caused by fructans in processed meat products is not well understood. It has been speculated that increases in hardness may occur as a result of fructans' ability to promote interactions between various components found within the matrices or the fact that fats are physically softer in comparison to inulin crystals (Cruz et al., 2010; Keenan et al., 2014). Nonetheless, it appears that fructans may indeed be able to partially replace fat in meat‐type products suggesting further work in this area would be highly beneficial to optimize product formulation. This is particularly important given that several of the meat products mentioned may be able to deliver a high enough dose of fructans to stimulate a beneficial response in the gut microbiome.

## 4 INULIN‐TYPE FRUCTANS AND SHORT‐CHAIN FRUCTOOLIGOSACCHARIDES AS A SUCROSE REPLACER

The main sugar used in the production of cake, biscuits, ice cream, meat products, jam, jellies, and fruit juices among others is sucrose (Clemens et al., 2016). Sucrose is primarily known for contributing a sweet taste, tempering bitterness, and acidity, along with acting as a bulking agent (Goldfein & Slavin, 2015). Beyond this, sucrose is also a precursor for flavor compounds and color via participation in both the Maillard and caramelization reactions (Hwang et al., 2011). In addition, it contributes to the brittle texture of candy and smoothness and creaminess of iced desserts via the melting of sucrose into its crystalline and amorphous form and reducing the rate of ice crystal formation during freezing, respectively (Clemens et al., 2016; Cook & Hartel, 2010).

Furthermore, sucrose is critical in the preservation of jams, jellies, preserved fruits, and even meat due to its water‐reducing activity (Goldfein & Slavin, 2015). The water‐binding capacity of sucrose also provides tenderness in baked products via competition, with starch and protein molecules for liquid components in batters and doughs, limiting gluten formation, and raising starch gelatinization temperature (Rodriguez‐Garcia et al., 2014). Sucrose also stabilizes cake batters and provides lightness by interactions with egg proteins, making the final batter more elastic (Pareyt et al., 2009). These interactions allow more air bubbles to be incorporated during creaming, preventing them from escaping during baking, and permitting expansion to occur (Gao et al., 2016). Thus, replacing sugar with a suitable alternative must be carefully considered. Table 2 summarizes the findings of ITF and scFOS as sugar replacers.

**TABLE 2:** Summarized findings of ITF and scFOS used as sucrose replacers in food products

Reference	Food product	Type of inulin	Level of sugar/Fructan replacement	Outcomes/Findings
Klewicki, 2007	Apple and blackcurrant juice Milk–peach–mango drink	Sc‐FOS was synthesized using enzyme preparation	1.5 g/100 ml	Under two‐stage processing (Stage 1: 95°C 30 s and Stage 2: 84°C for 10/20 min), the concentrations of scFOS tetramers and higher oligomers decreased by up to 70%–87% At pH 4.2, 95°C, and 30 s, 80% of scFOS was retained during the pasteurization of a milk–peach–mango drink
Duar et al., 2015	Prototype drink	Sc‐FOS, average 1‐kestose 33.8%, DP 3; nystose 50.1%, DP 4; and fructosyl nystose 11.6%, DP 5 (GTC Nutrition) Orafti® inulin ≥92% oligofructose, average DP 3–60	1% w/w	At pH 3, only between 35 and 50% of scFOS survived the pasteurization process At pH 4, scFOS was relatively stable with 80–90% of scFOS in a prototype drink being retained after the pasteurization process Over 90% of LC‐ITF survived the pasteurization at pH 3 with all inulin surviving the pasteurization process at pH 4
Glibowski et al., 2020	Apple Juice	(HP) Frutafit®TEX, average DP > 23, (NAT) Frutafit®IQ, average DP > 10 OF Orafti®P95, average DP > 4–5	0%, 2%, and 4 g	No significant differences were detected in reducing sugars throughout the course of storage No differences in sensory characteristics were detected between the control and all ITF‐containing samples Both native and HP ITF added into apple juice at 2% and 4% were unaffected via pasteurization at 100°C for 1 min at pH 3.68 ± 0.16
Aidoo et al., 2014	Chocolate	Rafteline®HP, average DP > 23	100%	Chocolate replaced with 100% ITF at 30% fat content matched the 0% inulin control in terms of hardness, as well as melting properties at 27%, 30%, and 33% fat content
Konar et al., 2014	Chocolate	Inulin (no specific details)	6%, 9%, and 12%	As levels of ITF fortification increased, the largest particle size (D90 value) increased, ranging from 39.80–44.49, 54.23–55.71, to 61.50–62.61 cm^3^/cm^2^ At 12%, ITF replacement as a mean partial size of 20 um was achieved at 3.5 h conching
Farzanmehr & Abbasi, 2009	Chocolate	Frutafit® IQ, TEX, CLR, average DP >5–7, > 23, 7–8	41.8 g/100 g	Sugar‐replaced ITF chocolate possessed similar levels of sweetness, hardness, and color compared to the full‐sucrose control A significant difference in melting rate and mouth coating was detected between the control and ITF‐replaced chocolate (*p* < .05)
Rodriguez‐Garcia et al., 2014	Cake	Frutafit®HD, average DP 8–13(9)	0%, 20%, 30%, 40%, and 50%	As ITF/sugar replacement increased, batter viscosity decreased (*p* < .05) At 50% sugar replacement, number of bubbles decreased and the size of the remaining bubbles increased Cakes at 30% and 40% ITF/sugar replacement recorded significantly greater weight loss (%) after baking (*p* < .05) As sugar replacement increased to >40%, cake height decreased significantly (*p* < 0.05) As sugar replacement increased, hardness decreased significantly (*p* < .05) Sugar replacement by oligofructose at 30% (C0‐30) did not show significant differences (*p* > .05) from the control cake Cakes made with 50% ITF/sugar replacement recorded lower overall intention to purchase scores compared to control cake
Gao et al., 2016	Muffins	Frutafit®IQ, average DP 5–7	50% and 100%	At 50% sugar replacement, muffins possessed similar texture compared to the control. In contrast, muffins made with 100% sugar/ITF replacement recorded significantly higher firmness and lower springiness values (*p* > .05)
Tsatsaragkou et al., 2021	Cake and biscuits	Orafti®HSI, average DP unknown Fibruline® Instant, average DP approx. 10	0% and 30%	Orafti® HSI produced similar batter viscosity compared to the control. Whereas Fibruline® Instant produced significantly higher batter viscosity (*p* > .05) Sugar‐replaced ITF sponge cake retained significantly higher moisture content compared to the sugar control after baking (*p* > .05) The springiness of ITF‐supplemented sponge cakes was significantly lower compared to the control (*p* > .05) Orafti® HSI sponge cake exhibited significantly lower (*p* < .05) firmness values compared to the control. However, Fibruline® Instant‐supplemented sponge cake reported significantly higher (*p* < .05) firmness value Several sensory attributes including color, dry appearance, dry to touch, springiness, and aroma were all significantly impacted by sugar/ITF replacement in cake (*p* < .05) ITF‐supplemented biscuit dough was significantly harder and possessed higher levels of dough stickiness compared to the control (*p* < .05) No differences in color were detected between control and ITF/sugar‐replaced biscuits (*p* > .05) Significant differences in the density of crumb were detected between ITF‐supplemented biscuits and the control (*p* < .05) Several sensory attributes including vanilla flavor and crunchiness were scored significantly lower compared to the full‐sugar control (*p* < .05)

### 4.1 Fruit juices

Fruit Juices and sugar‐sweetened fruit juices are highly desirable products among all age ranges due to their pleasant sensory characteristics and health benefits including being a source of several beneficial vitamins and minerals such as vitamin C, as well as potentially other useful compounds such as pectins and phenolic acids (Gomes et al., 2017). However, due to the presence of high levels of sugars and organic acids, fruit juices play a major role in the development of dental caries (Liska et al., 2019). As well as being a contributor to an increased risk of diabetes (Xi et al., 2014). Thus, to reduce the onset of dental caries and diabetes, replacing sugars with alternatives has become of increasing interest to the fruit juice industry.

The addition of prebiotics including fructans to fruit juices at face value seemingly makes logical sense as they cannot undergo digestion in the GI tract, including in the oral cavity, due to lack of β‐fructosidases needed to hydrolyze β‐(2–1) glycosidic linkages (Capuano, 2017). Yet, the addition of fructans to fruit juices represents a major technical challenge for manufacturers due not only to low pH but also the temperatures and pressures used during the pasteurization process (Fonteles & Rodrigues, 2018).

Several attempts have been undertaken to replace sugar with fructans in fruit juices. Klewicki (2007) exposed apple and blackcurrant juice drinks containing scFOS at 1.5% (1.5 g/100 ml) to two‐stage processing (Stage 1: 95°C 30 s and Stage 2: 84°C for 10/20 min) noting that concentrations of scFOS tetramers and higher oligomers decreased by up to 70%–87%. Furthermore, it was recorded that a significant portion of the tetrasaccharides and higher scFOS fraction present in the juice were degraded to trisaccharides, subsequently increasing the number of trimers that could be hydrolyzed to di‐ and monosaccharides, respectively.

However, it could be argued that these conditions do not directly reflect those used in modern fruit juice production. The same authors also reported that under less extreme processing conditions, similar to those used in the food industry (pH 4.2; 95°C 30 s), 80% of scFOS was retained during the pasteurization of a milk–peach–mango drink: a finding mirroring those of (Duar et al., 2015) who recorded that at pH 4, scFOS was relatively stable with 80%–90% of scFOS in a prototype drink being retained after the pasteurization process had been completed. Interestingly, Duar et al. (2015) did detect substantial differences in the rates of hydrolysis between scFOS and LC‐ITF, with over 90% of LC‐ITF surviving the pasteurization at pH 3. These findings are in line with those reported by (Glibowski et al., 2020), who noted that the levels of both native and HP inulin added into apple juice at 2% and 4% were unaffected by pasteurization at 100°C for 1 min at pH 3.68 ± 0.16.

The degradation of fructans in fruit juices results from complex interactions among chemical structure, pH, temperature, and pressure, whereby the higher the temperature, the more elevated the pressure and the lower the DP. Lower pH (pH 4 being the critical limit) also induces the degradation of fructans (Fonteles & Rodrigues, 2018; Klewicki, 2007). Any losses in fructan as a result of pasteurization can be overcome or minimized by either maintaining pH at or around >4, storing at <7°C, and/or utilizing LC‐ITF in production. Fructans would seem to be a suitable ingredient in acid‐based fruit drinks.

### 4.2 Chocolate

Chocolate production is a highly complex physical and chemical process where chocolate can be defined as a suspension/dispersion of nonfat and fat ingredients including cocoa powder and sugar in a Newtonian fluid (Konar, 2013; West & Rousseau, 2018). The quality of chocolate is judged on its snap, melting qualities, aroma, flavor, and particle size distribution with each of the ingredients mentioned above affecting the quality of the final product (Glicerina et al., 2014).

The majority of sugar used in chocolate production comes in the form of sucrose and plays several major roles including tempering bitterness, contributing to the formation of aroma compounds, and affecting flow quality and nucleation (Svanberg et al., 2011; Torres‐Moreno et al., 2012), as well as acting as a bulking agent making up between 30 and 50% of the final product (Aidoo et al., 2013; Gutierrez, 2017). During production, mixtures of cocoa, sugar, and fat are refined via a procedure commonly termed conching (Prawira & Barringer, 2009). Conching is a process where shear and heat are applied to two phases: dry and wet over a 16‐ and 48‐h period resulting in the production of one final liquified mass (Konar, 2013). The process of conching is critical in the production of fine quality chocolate as it results in the reduction in particle size directly impacting final texture, viscosity, and flavor (Schumacher et al., 2009; Torres‐Moreno et al., 2012).

To obtain fine‐textured chocolate, a reduction of 90% of particles to below 20–25 μm must be achieved (Gutierrez, 2017; Oba et al., 2017). When particles do not reach these values, chocolate is frequently described as coarse and gritty, with chocolate possessing particle sizes >35 μm being labeled as unpalatable (Kalic et al., 2018; Schumacher et al., 2009).

Particle size is directly related to the specific surface area, with particles seemingly becoming more spherical as specific surface area increases, resulting in the broadening of particle size (Glicerina et al., 2014). The relationship between particle size and specific surface area ultimately influences both yield stress and fracture rate, directly impacting the phase transition state, from solid at room temperature to liquid at body temperature (Glicerina et al., 2014; Konar et al., 2014). This implies that the replacement of sucrose with alternatives in chocolate must be carefully considered to optimize the ratio between the number of coarse and fine particles to help reduce the potential impact that replacement of sugar may have on final rheological and sensory properties (Konar, 2013).

Konar et al. (2014) investigated the effects of the addition of ITF (no specific details) at 6%, 9%, and, 12% (w/w) on final particle size. The authors noted that, as the level of ITF fortification increased, the largest particle size (D90 value) increased, ranging from 39.80–44.49, 54.23–55.71, to 61.50–62.61 cm^3^/cm^2^, respectively. Yet, despite the largest particle size increasing, it was possible to produce a chocolate with a mean particle size of 20 μm at 12% ITF fortification even at the lowest conching time (3.5 h). Adding to this, Farzanmehr and Abbasi (2009) noted that in milk chocolate, in which sucrose had been replaced with ITF (Frutafit® IQ, TEX, CLR) at 41.8 g/100 g chocolate, the chocolate had similar levels of sweetness, hardness, and color compared to the full sucrose control. However, the addition of ITF resulted in alterations to the melting characteristic as detected by the sensory panelists, a finding in line with those reported by (Aidoo et al., 2014) who recorded that chocolate replaced with 100% ITF (Rafteline®HP) could replicate a level of hardness comparable to that of the 100% sucrose control when the fat level was around 30%. Furthermore, the same authors noted that at 100% sucrose replacement, the melting properties remained unaltered compared to the full sucrose control even when fat was increased from 27%, 30%, to 33%. Although, as the melting rate was determined by textural analyzer, making any comparisons with the results detected by (Farzanmehr & Abbasi, 2009) cannot be undertaken.

Nevertheless, it seems likely that ITF can be successfully used as a partial replacement for sucrose in chocolate without a loss of physical and sensory properties. In order to optimize product quality, however, it is clear that a greater understanding of the functionality of bulking agents is still required.

### 4.3 Baked products

Baked products, notably cakes and biscuits, are well renowned for their high sucrose content contributing to their sweetness, snap, crispness, aeration, browning, and shelf life (Pareyt et al., 2009; Rodriguez‐Garcia et al., 2013). Sucrose also elevates starch gelatinization and egg white protein denaturing temperature, allowing for the development of a finer‐textured cake crumb and greater retention of final moisture content (Gao et al., 2016). ITF, due to its polydisperse nature consisting of both LC‐ITF and OF, represent the realistic alternative to sucrose in cakes and biscuits because of their physically different properties. LC‐ITF provides water‐holding and occluding properties, while OF mimics the qualities of glucose syrup or sucrose providing a source of sweetness (Martins et al., 2019).

The replacement of sucrose in cakes with OF was investigated by Rodriguez‐Garcia et al. (2014) ranging from 0 to 50%. The authors noted that sponge cakes replaced with 30% ITF gave similar scores in terms of color, appearance, texture, taste, and overall acceptability compared to the full‐sugar control. However, there was a noticeable loss in cake height, likely resulting from the decline in batter viscosity and a reduction in the number of occluded air bubbles within the final batter. These alterations become increasingly apparent at 50% sucrose/ITF replacement, with noticeable differences in textural (cohesiveness, springiness, and chewiness) and taste characteristics (*p <* .05).

From this, it could be inferred that the degree to which textural and sensory characteristics are affected is highly dependent on the level of sucrose/fructan replacement. On this basis, Rodriguez‐Garcia et al. (2014) suggest that the replacement of sucrose with 30% ITF appears to be an optimal level of sucrose replacement before substantial changes in sensory characteristics begin to become increasingly apparent. However, in contrast, Gao et al. (2016) noted that muffins with 50% ITF/sucrose replacement recorded similar values in terms of firmness and springiness compared to the control, inferring that the level of sucrose replacement with OF may not be the only determining factor.

Another contributing factor is the DP of inulin used during production, with Tsatsaragkou et al. (2021) documenting that higher DP inulin (Fibruline® Instant) resulted in a more vicious cake batter (*p* < .05). This leads to a less homogenous cake structure and greater perceived levels of mouth coating and dryness. While the same authors noted in biscuits produced using lower DP inulin (Orafti® HSI) as a sugar replacer that final biscuits were softer and less crunchy compared to full‐sugar control.

In addition to the level of sucrose/fructans, replacement, and DP of fructans used during production, variations in results may have also occurred due to differences in ingredient composition, with a key difference being the initial level of fat used in production. In this regard, Rodriguez‐Garcia et al. (2014) noted in cakes possessing higher initial levels of fat, where sucrose was replaced with OF, a more viscous batter was able to be obtained up to 30% sucrose/ITF replacement. As a more viscous cake batter allows for more air to be occluded during creaming, this results in a more pronounced rise during baking producing a lighter and more acceptable final cake/muffin (Psimouli & Oreopoulou, 2013; Wilderjans et al., 2013). Yet interestingly, Rodriguez‐Garcia et al. (2014) also recorded that sponge cakes in which fat and sugar had been replaced with 50% and 30% OF had good overall acceptability compared to full‐fat and sugar control.

This suggests that it may be possible to produce an acceptable sponge cake while reducing both fat and sugar simultaneously, but further work in this area is still required in order to optimize product formulation.

## 5 INULIN‐TYPE FRUCTANS AND SHORT‐CHAIN FRUCTOOLIGOSACCHARIDES AS TEXTURE MODIFIERS

The textural aspect of food is an extremely critical factor regarding consumer acceptance (Tomadoni et al., 2018). Manufacturers are frequently targeted with reformulating products due to changes in consumer trends and increasing health concerns (Unal & Akalin, 2013). Dairy products including yogurt, spreadable cheese, and custard‐based desserts are among the most common foods subjected to reformulation due to their high levels of saturated fat and/or sugar (Akin et al., 2007; Gonzalez‐Tomas et al., 2009; Karimi et al., 2015). However, the removal of fat and/or sugar results in a loss of textural properties, leading to a decline in consumer satisfaction (Gonzalez‐Tomas et al., 2009).

One way to improve and maintain the texture of reduced‐fat and sugar food is via the use of viscosity modifiers and hydrocolloids including xanthan, guar, and gellan gum (Saha & Bhattacharya, 2010). Yet, despite these texture modifiers and hydrocolloids proving to be effective in their ability to modify product rheology, they do not provide any additional nutritional benefits. It is on this basis that fructans, in particular ITF, represent a realistic alternative to viscosity modifiers due to their ability to form viscous and stable gels along with providing a much needed source of fiber (Meyer et al., 2011).

As the addition of ITF increases beyond 10%, changes in product rheology become increasingly apparent with the formation of an extremely thick solid‐like gel (Karimi et al., 2015), whereas above 20%, the gels retain a fat‐like texture, with increased levels of firmness. The ability of ITF to form gels with fat‐like characteristics results from the formation of microcrystals and aggregates, which can occlude a significant amount of water (Tarrega et al., 2011). The crystallization and aggregation of ITFs are highly complex and the rate is determined by a combination of several factors including the type, concentration, and DP of the ITF, as well as the rate of shear, temperatures used in production, and, critically, the food product in question (Torres et al., 2010).

One of the most promising applications of ITF to modify product rheology is the production of milk‐based dairy desserts. Tarrega et al. (2011) supplemented both skimmed‐ and full‐fat milk custard with either short‐chain (DP 2–10), LC (DP > 23), native inulin (DP 30%–40% < 10% and 60%–70% DP > 10), and a mix of short and LC‐ITF, all at 7.5%, looking for changes in rheological properties and microstructure. The results of this study showed that ITF aggregates did not form in the presence of short‐chain inulin, whereas the presence of inulin aggregates was detected in both skimmed‐ and whole‐milk custards made using either LC, native, or mixture of ITF, consequently resulting in a more thixotropic, consistent, and elastic final product. Yet, differences in the rate of changes in rheological properties and aggregate formation were seen between different types of ITF (LC‐ITF > mix of SC and LC > native) with these effects being more pronounced in skimmed‐milk samples than whole‐fat milk samples, respectively.

A finding confirmed by both Gonzalez‐Tomas et al. (2009) and Torres et al. (2010) is that the addition of either short‐chain, long‐chain, or native inulin at 7.5% to milk‐based desserts modified the rheology of the final product. However, alterations to final rheology were more pronounced in milk‐based desserts made with skimmed milk compared to whole milk. In addition, Gonzalez‐Tomas et al. (2009) also tested the effects of the addition of ITF to dairy based‐desserts on sensory quality. The authors, noted that while both skimmed‐milk and whole‐milk samples containing LC‐ITF were perceived to have the same levels of creaminess, dairy‐based desserts made with LC‐ITF were perceived to be rougher. Dairy‐based desserts made with the addition of short‐chain or native inulin and skimmed milk were evaluated as less rough and were also associated with higher levels of sweetness, vanilla odor, and flavor.

The differences in the level of thickness detected between whole‐ and skimmed‐milk dairy‐based desserts made with ITFs are complex and dependent on the amount of initial fat present and the type of ITF used in production (Gonzalez‐Tomas et al., 2009). The fat content of skimmed milk vs. full‐fat milk is roughly 1.7% vs. 3.5% (McCarthy et al., 2017). The lower abundance of fat in skimmed milk allows a higher number of larger ITF crystals and aggregates to form resulting in the formation of a more cohesive and stable network due to an increased ability to hold onto water (Torres et al., 2010). Levels of roughness detected in dairy‐based desserts appear to be highly dependent on the DP of the ITF used in production. It is frequently reported that in dairy‐based desserts made with OF or SC‐ITF, lower levels of roughness are detected (Bayarri et al., 2011; Tarrega et al., 2011; Torres et al., 2010), whereas dairy‐based desserts made with LC‐ITF are perceived to be rougher (Bayarri et al., 2011; Gonzalez‐Tomas et al., 2009). The differences are likely to occur as a result of an increase in the aggregation and particle size of inulin crystals.

The perceived level of roughness is also dependent on the rate of shear and thermal production methods used. At low shear, the lower level of disruption to the matrices allows the formation of larger crystals and aggregate clusters to occur (Bayarri et al., 2011; Torres et al., 2010). While in contrast, at high shear, better dispersion of these aggregates can be achieved. However, it has been reported that even at high shear, compared to a thermally induced gel, both larger particles and broader particle distributions are still detected. Via the repeated application of high shear throughout the production process, a reduction in particle size can facilitate the formation of a finer network (Mensink et al., 2015). With optimization of the manufacturing process, production of a low‐fat dairy‐based dessert with acceptable or improved rheology and sensory properties using either or a mixture of both short‐ and long‐chain ITF at 7.5% (w/w) can be achieved.

## 6 FUTURE WORK AND CONCLUDING REMARKS

It is clear from this review that both ITF and scFOS can act successfully as both fat and sugar replacer as well as a viscosity modifier in several different food products ranging from ice cream to bread, cake, shortbread biscuits, fruit juices, and meat. Yet, the degree to which fat and sugar can be successfully replaced with ITF and scFOS is highly dependent on the product formulation and the methods used during production. Therefore, future work should focus on refining both processing parameters and product formulation to optimize the level of supplementation to ensure consumer quality is met. Furthermore, while several studies have suggested that degradation of ITF and scFOS can occur during production, it remains unclear whether these alterations are substantial enough to alter their prebiotic efficacy, and thus should also be the focus of future work.

## ACKNOWLEDGEMENTS

Peter Jackson would like to thank Prof Bob Rastall and Dr Anisha Wijeyesekera for their support and feedback in writing this manuscript.

## CONFLICT OF INTEREST

None to declare.

## Data Availability

No data is available because it is a review article.
